# How can funding for nature-based programmes impact health inequalities? A realist protocol

**DOI:** 10.1186/s13643-025-02938-5

**Published:** 2025-10-24

**Authors:** Hannah Forbes, Benedict Wheeler, Jonathan Reeves, Rebecca Lovell

**Affiliations:** 1 European Centre for Environment and Human Health, University of Exeter, Peter Lanyon Building, Penryn, TR19 9FE UK; 2 The Wildfowl and Wetlands Trust (WWT), Slimbridge, Gloucester, GL2 7BT UK

**Keywords:** Realist synthesis, Realist review, Nature-based programmes, Green prescribing, Blue prescribing, Health inequalities, Asset-based approaches, Funding mechanisms

## Abstract

**Background:**

Nature-based programmes facilitate access to group-based structured activities that take place in the natural environment. These programmes have been found to improve the health and wellbeing of participants. However, downstream individualised interventions can be associated with intervention-generated inequalities. There is a concern that if nature-based programmes are not designed and delivered appropriately, they could worsen health inequalities. Many nature-based programmes are either reliant on charitable funders or public sector funding. It is likely that the source of funding will have consequences for who the programme is for and who is able to access the programme. However, there is a lack of robust understanding of how the funding system contributes to the outcomes of nature-based programmes.

**Method:**

The aim of this project is to explore what is known about the impact funding of nature-based programmes can have on health inequalities, primarily through impacts on the targeting of, and recruitment to the activities. Secondary and primary data will be used to develop, refute and refine programme theory. Initial programme theories will be developed through scoping the literature and theory gleaning qualitative interviews. These theories will be tested and refined through a realist review of evidence and further qualitative realist testing interviews.

**Discussion:**

This project aims to inform practical strategies and frameworks (underpinned by programme theory) to help programme funders, designers and implementers understand how to design and deliver their programmes as equitably as possible.

## Background

The positive impact of nature on health and wellbeing is well documented, with evidence suggesting benefits for both physical and mental health [1–3]. Research also shows that proximity and use of green and blue spaces are associated with lower socioeconomic inequalities in health and wellbeing [4–6] and time spent in nature appears to be more beneficial to health for people who live in more disadvantaged areas or face higher levels of structural disadvantage [7, 8]. The potential for green and blue spaces to contribute to health equality within the constraints of the existing unequal distribution of resources is known as the ‘equigenesis hypothesis’ [4, 9]. The equigenesis hypothesis argues that policies and programmes that enable access and use of green and blue spaces may help to reduce health inequalities [5].

In the UK, policy emphasis on asset-based approaches to health care [10] and the link between nature and health [3] has contributed to the development and funding of nature-based programmes (NBPs). NBPs range from infrastructural interventions such as implementing green and blue spaces in urban environments that aim to bring nature to people [11] to programmatic interventions such as care farming to bring people to nature [12]. This paper focuses on programmatic interventions and defines NBPs as programmes that aim to enable health and wellbeing benefits through providing access to specifically designed, structured and facilitated group-based activities that engage people with nature [13]. A wide range of activities or strategies can be employed as part of these programmes such as gardening and horticulture; green and blue exercise; conservation activities; and nature-based therapies [14]. Previous reviews of programmatic NBPs have found that they can have a positive impact on the health and wellbeing of participants [14–16]. However, there is a concern that NBPs may not be accessible to everyone [17] and that the associated health benefits may be socially patterned. Researchers have highlighted that ‘downstream’ individualised interventions can make health inequalities worse as they do not address the structural causes of unfair differences in health [18–20]. On top of this, individualised interventions that focus on behaviour change have been shown to disproportionately benefit less disadvantaged groups of people [21, 22]. This is reinforced by findings from Boyd et al. [23] who found that infrequent visitors to green and blue spaces tend to be of lower socioeconomic status, of ethnic minority status, living in relatively disadvantaged areas, and in poor health. In the context of growing health inequalities in the UK [24], the disparity in the use of green and blue spaces between population groups is concerning because the groups of people identified as being less likely to visit green and blue spaces also are more likely to experience poor health and have also been identified as being more likely to benefit from accessing green and blue spaces [4, 25].

There are physical, psychological, social and cultural barriers and enablers in accessing NBPs. A major physical barrier is that nature-based organisations, which are often involved in the design and delivery of NBPs, are more likely to be located in more affluent areas of the UK [26]. This reflects Hart’s inverse care law whereby people who are most in need of health and social care are the least likely to receive it [27, 28]. Other physical barriers include lack of facilities such as toilets and benches [29]. Psychological, social and cultural barriers include differing cultural experiences of green and blue spaces, motivational factors, potential for stigmatisation, language restrictions, and awareness of the programme and its benefits [30–32]. Further barriers include complex lives with, for example, caring duties, multiple jobs or other commitments leaving little space for travel to and attendance of an NBP. If NBPs are not designed to consider the barriers that some individuals and communities face, the programmes are likely to result in inequitable uptake and adherence and therefore benefit.

The funding system for NBPs is complex with multiple funding models and varying stakeholder priorities [33]. Funding to deliver NBPs typically comes from charitable organisations, corporate donations, self-funding and direct commissioning from NHS or local authorities [16]. The funding allocated to providers tends to be short term and prioritises novelty rather than the sustainability of the programme [16, 34, 35]. A survey of green activity providers in England found that most providers believe that changes to the funding system would enable NBPs to benefit more people equitably [34]. The current funding system has consequences for the type of programme that is being delivered and who can access it. This can happen through conflicting priorities between the funders and providers of the programme. Often, NBPs rely on funding that prioritises national strategy and policy but not necessarily local needs [16]. This results in a mismatch between demand and supply of NBPs. Lack of continuity in funding means that providers have to invest resources to apply for further funding which can impact programme delivery [16]. The process of seeking new funds can also have a substantial impact on smaller grassroots providers who may have less capacity to constantly seek further funding [16]. Smaller providers have also raised concerns about biases towards larger organisations in commissioning processes [35]. Short-term funding cycles can also impact stakeholder ‘buy in’ to the NBP, whereby local stakeholders are less willing to invest in the NBP, as knowledge and connections to local community groups are lost once the funding ends [36]. The current funding system places the emphasis on nature-based providers (most of which are located in less structurally disadvantaged areas [26]) to apply for funding to deliver programmes. This could further worsen local health inequalities as access to NBPs becomes dependent on having the appropriate community-based providers to champion NBPs [16]. On top of this, there may be less available funding in disadvantaged areas as local government funding is lower per person compared to more advantaged areas [37].

The ability of NBPs to address some of the physical, psychological, social and cultural barriers in access to NBPs is likely to depend on the priorities of the funders and providers of the programmes. NBPs can be designed and delivered to meet the needs of specific groups of people, or they can be preventative nature-based activities aimed at the general population. A targeted NBP is designed to address specific needs or engage a specific community (for example according to health condition or demographics). However, NBPs are not always explicit in their offer [16], and often there is overlap between targeted and non-targeted programmes as people attending activities for the general population may also have a health or social care need. There is uncertainty whether specifically targeted programmes or programmes for the general population are more appropriate for addressing health inequalities [38–40]. Marmot proposes that programmes should be implemented for the whole population but with a scale that is proportionate to need [19]. However, identifying appropriate support that is proportionate to need is complex and may not align with the priorities of funders. The aims and approaches of NBPs are likely to differ between targeted and non-targeted programmes and this could have an impact on the barriers people face in accessing the programme. Barriers and enablers to NBPs also emerge through the process of recruiting the targeted communities or individuals to the NBP. Recruitment strategies include providing transportation, community engagement, cultural adaptations, providing information about the NBP and self-referral. Transportation to NBPs, for example, has been found to be associated with participants enrolling in the programme [41]. Knowledge of the potential benefits of the programme and what is expected of participants has also been shown to impact programme uptake [41].

**Fig. 1:**
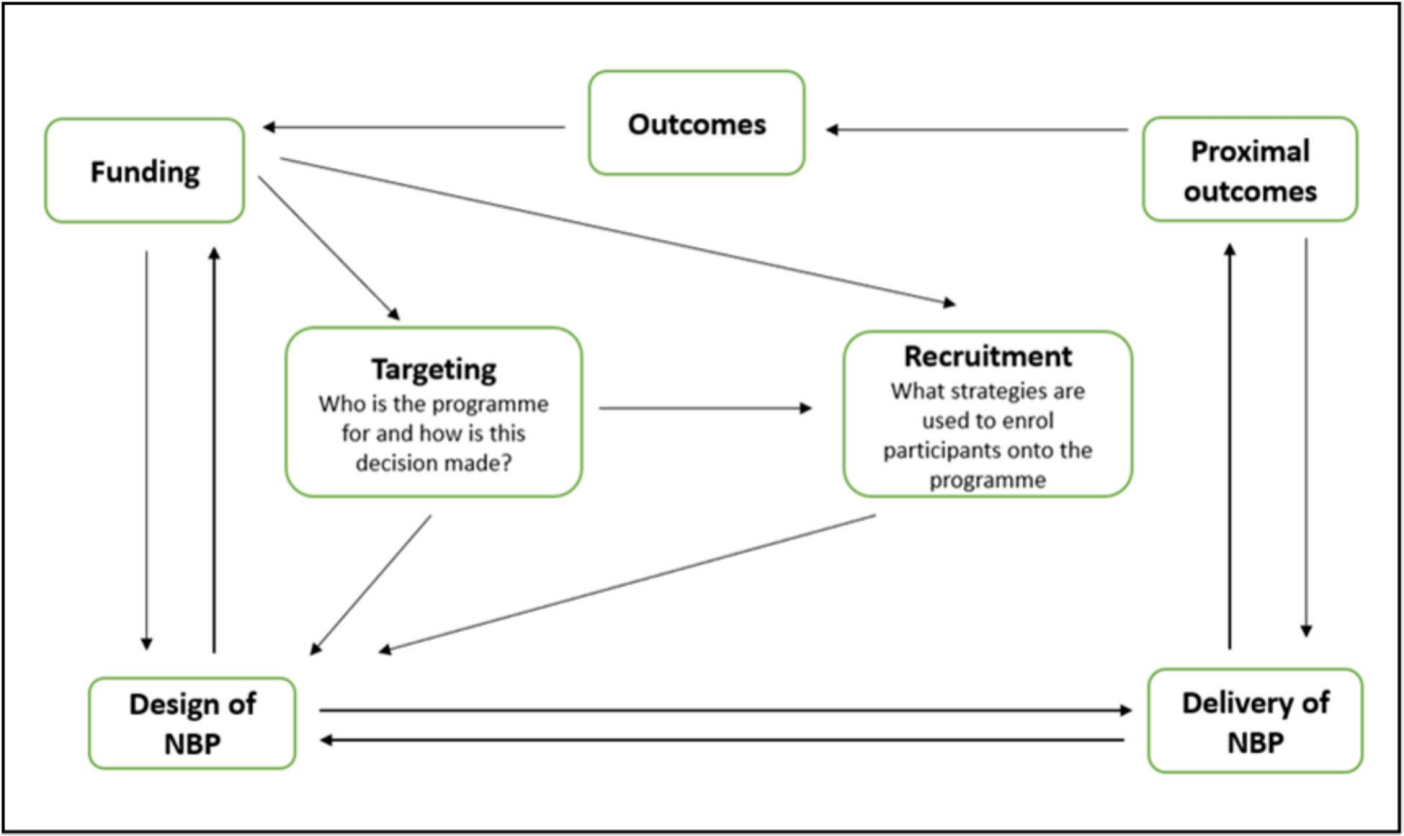
The processes associated with the delivery of NBPs

The delivery of NBPs involves a series of complex processes that require multiple stakeholders (participants, funders, voluntary sector workers, programme staff and facilitators) to play an active role in the programme (Fig. 1). The funding for NBPs will impact who the programme is for and how the targeted recipients are recruited.

## Research aims

Recognising how funding for NBPs can impact who is able to attend and benefit from the programme is vital to improving the accessibility of the programmes and, therefore, their impact. To explore the complexity inherent in the funding system and delivery of NBPs, realist approaches will be used. Realist approaches are theory-driven and focus on the interaction between causal mechanisms, contexts and outcomes. Pawson and Tilley [42] argue that programmes alone do not bring about the observed outcomes, but outcomes are generated through the stakeholders’ response to the resources, ideas and practices that the programmes introduce. According to Pawson and Tilley, for any outcome that is empirically observed, there are unobserved causal forces (known as mechanisms) that become activated in specific contexts. The purpose of realist research is to produce a refined and tested programme theory—ideas about how a programme causes the intended or observed outcomes [43].

The purpose of this study is to conduct a realist review of evidence on the funding and targeting strategies associated with NBPs. The overarching research question is: What is known about the impact funding of NBPs can have on health inequalities, primarily through the targeting of and recruitment to the activities? To approach this, initial programme theories shown in Appendix 1 will be tested. To develop and refine the programme theories, a realist analytical approach will be applied to data drawn from a range of sources, including primary data, non-academic ‘grey’ literature and empirical evidence.

### Objectives

1. To use a theory-driven approach to identify factors that influence how the funding of NBPs impacts who the programme is for and how people access the programme.
2. To produce refined programme theories of causal mechanisms that become activated in specific contexts to produce the observed outcomes.
3. To develop practical strategies and frameworks (underpinned by programme theory) that help programme funders, designers and implementers understand how to fund, design and deliver NBPs as equitably as possible.

## Methods

### Research plan

**Fig. 2:**
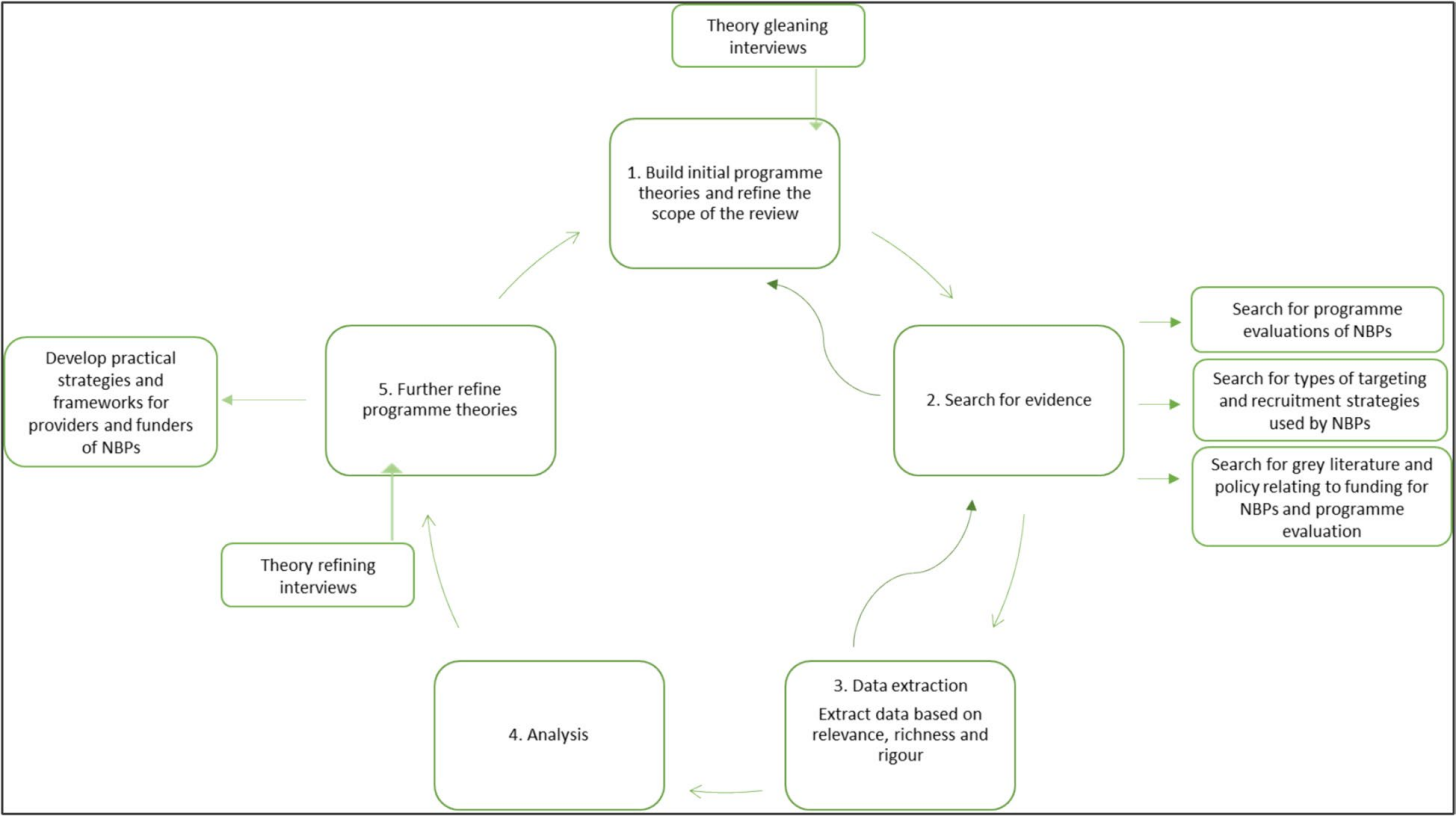
Diagram of the realist review process

This realist review will develop and refine programme theory through a combination of secondary and primary data collection methods. Inclusion of primary data in the research will give deeper insight into possible causal mechanisms that may not be apparent in the literature and will give greater strength to stakeholder voices throughout the review. According to Pawson [44] programme theory is refined, confirmed or abandoned through the relevance and richness that can be obtained through a mixed-method strategy. Other realist projects that have used primary data collection alongside a realist review include Maidment et al. [45] and Cooper et al. [46]. The review will be conducted and reported according to the RAMESES (Realist and Meta-narrative Evidence Syntheses: Evolving Standards) publication standards for realist reviews [47, 48] and will follow the task and time template outlined by Pawson [49] for realist reviews. A diagram of the review process is outlined in Fig. 2. Due to the exploratory nature of realist research, the review will be iterative allowing for the refinement of programme theory to happen throughout the process. Therefore, stages may overlap or proceed in parallel as the review progresses.

### Step 1. Build initial programme theories and refine the scope of the review

The first stage will begin with developing and gleaning initial programme theories which aim to explain how the funding of NBPs impacts on the targeting and recruitment strategies used by the programme. These initial programme theories will then be iteratively refined throughout the review process. Initial programme theories will be identified and developed through an initial scoping of the literature, theory gleaning interviews and feedback from a stakeholder advisory group.

#### Scoping review

The initial scoping review will focus on literature regarding intervention-generated inequalities, targeting of NBPs and funding for NBPs. This initial search will be exploratory and use informal methods such as snowballing and citation tracking [50].

#### Theory gleaning interviews

Exploratory theory gleaning interviews will be conducted alongside the scoping review. The purpose of these interviews is to understand the process of applying/allocating funds for NBPs and how that process can impact who the programme is for and how people engage with the programme. Purposive sampling will be used to recruit 15 interviewees from a range of organisations involved in the funding and delivery of NBPs in England including large nature-based organisations, funders of NBPs, local government and small-scale providers. Key interviewees will be identified through scoping grey literature, immersion into the field and snowballing. Potential interviewees will be contacted via email to invite them to participate in the research project. Due to the budget constraints of the project, interviewees will not be reimbursed for their time. Participant information sheets will be provided and informed consent obtained prior to the start of the interview. The interviews will last between 30 and 60 min and will take place online using Microsoft Teams. Interviews will be audio recorded and manually transcribed. All data will be anonymised. Data from the interviews will help develop initial programme theories to be tested throughout the review.

#### Stakeholder advisory group

A stakeholder advisory group of relevant professionals (those involved in the funding, design and delivery of NBPs) will be convened. The stakeholder group will help to refine and prioritise the initial programme theories and highlight any key literature that is missing from the scoping review.

### Step 2. Search for evidence

The purpose of this stage is to further refine the initial programme theories through searching for relevant literature. The searches conducted in this stage will aim to identify empirical and theoretical literature to provide data that is relevant to the programme theory under examination. There will be 3 separate searches for evidence. The first search will focus on programme evaluations of NBPs. This search will seek to identify how the funding system surrounding NBPs can impact on the observed outcomes of NBPs. The second search will focus on the targeting and recruitment strategies associated with NBPs. The third search will look for grey literature and policy relating to funding for NBPs and programme evaluation. As there is no traditional hierarchy of evidence in realist reviews, a wide range of literature and documentation that is relevant to the initial programme theories will be sought [47]. Therefore, searches will focus on both academic databases and sources of grey literature. Some of the databases used to search for academic literature will include Medline, Embase, Web of Science, Scopus and PsychInfo. Grey literature will be identified through Google, citation tracking and from realist theory gleaning interviews conducted in stage one. Forward and backward citation searches will also be used. Programme theory will refine throughout the search process. Search terms will be derived from the prioritised initial programme theories and key publications identified through the scoping review. The search terms will relate to NBPs; the funding system surrounding NBPs; the types of targeting and recruitment strategies used by the programmes; and potentially the communities of interest (e.g. disadvantaged, marginalised, structurally disempowered).

### Step 3. Data extraction and quality appraisal

Results from the searches will be recorded using the Preferred Reporting Items for Systematic Reviews and Meta-Analysis (PRISMA) tool [51].

#### Study selection

Results from the database searches will be exported to EndNote [52] and deduplicated. The final results will be uploaded to Rayyan [53] for screening. HF will screen the titles and abstracts. To capture literature that has relevance to the initial programme theories, the title and abstract screen of the literature will be intentionally inclusive [54]. Full text screening will be undertaken by HF. BL will also screen a random 10% sample at both the title and abstract stage and full text screening stage. Any disputes will be resolved through discussion with the whole research team and refinement of the inclusion/exclusion criteria. Evidence identified through the searches will be assessed against the following inclusion criteria:

#### Population inclusion criteria

In all searches, evidence will be included relating to populations or communities associated with NBPs. Studies will include all age groups. At the full text screening, evidence relating to NBPs delivered in UK settings will be prioritised. However, other contexts may be included if they can fill any gaps in the literature.

#### Intervention inclusion criteria

First search: NBPs will be defined as funded and structured group-based activities and programmes that use exposure to nature as a tool to improve health and wellbeing. NBPs that meet these criteria will be included.

Second search: Targeting strategy will be defined as the process of clarifying who the NBP is for. The recruitment strategy will be defined as the process of reaching out and enrolling the targeted communities or individuals to the programme.

Third search: In the third search for evidence, the inclusion criteria will widen to include evidence that can further clarify causal pathways. This evidence can be drawn from wider literature that may not directly relate to NBPs. For example, evidence relating to community-based programmes and downstream individualised behavioural change interventions might be included if it is deemed relevant to the causal pathways under examination.

#### Comparator

No comparator criteria will be used to assess the evidence for inclusion.

#### Outcomes

A range of programme and individual outcomes relating to the impact that the funding system can have on the NBP, targeting and recruitment will be included. These include measures of health and wellbeing, demographics of participants attending, attrition from programmes, recruitment issues, engagement with the programme and uptake of the NBP. These outcomes are likely to be refined throughout the review process.

#### Study design

For realists, there is no traditional hierarchy of evidence [55]. Therefore, all study types will be sought. On top of this, other sources of evidence that may be relevant to the programme theory will be included, such as funding calls, evaluation reports, opinions/commentary and policy documents.

### Screening process

#### Quality assessment

At the full text screening stage, documents will be reviewed based on their relevance, richness and rigour (Table 1). The relevance of evidence may change as programme theories evolve. Consequently, initial search results and excluded evidence will be retained as they may prove to be relevant further on in the realist review.

**Table 1:** Relevance, richness and rigour

Relevance	Does the document contribute to the programme theory being refined? · Does the document describe who the NBP is for and why? · Does the document describe the priorities of funders? · Does the document describe the process through which the NBP was designed? · Is the document relevant to a UK setting?
Richness	Is the document able to explain how a programme is supposed to work? · Does the document include any theoretical underpinnings · Does the document provide sufficient detail about the contexts of the NBPs to allow for transferable findings?
Rigour	Are the methods employed trustworthy and credible? Do the outcomes support the conclusions drawn? Guiding questions from Morton et al [56] will help to transparently assess the rigour of the evidence. These questions include: · Is the data likely to be biased? · Is the subject/data dealt with critically? · Is it safe to generalise from this data?

The data extraction process will be refined throughout the review based on discussions with the review team and the emerging themes from the included studies. Programme theory will be used to develop a data extraction sheet to help structure relevant data and insights. Data extracted will include the study aims, design and methods, study participants, study outcomes and data relating to the programme theories.

To avoid missing any ‘nuggets of wisdom’ within the data, analytical journalling will also be used. A journal entry for each article will be written with the broad question ‘what is important about this article for the overall analysis?’ This process may uncover new ideas that can be refined through data analysis.

To add to the transparency of the synthesis process, the reasoning behind what data is extracted will be reported. The results of data extraction will be regularly discussed with the research team to ensure consistency and reduce bias when refining programme theories.

### Step 4. Analysis

The aim of the synthesis is to build theory relating to how the funding for NBPs can impact health inequalities, primarily through targeting and recruitment strategies used by the NBPs. This will be done by identifying how observed outcomes are generated through mechanisms that are activated in specific contexts. Recurring patterns across the data will be sought to understand what contexts activate the mechanisms. These recurring patterns will help to refine and refute the initial programme theories.

The analytical process will be iterative allowing for data analysis to be conducted while data is still being collected. Data analysis will use realist logic and will seek to understand how the data informs the development and refinement of programme theory and what refinements (if any) need to be made. There will be two approaches to analysis: prevalence of insight analysis and causal analysis. Initially, we will explore the prevalent initial programme theories that explain how the funding of NBPs impacts on targeting and recruitment strategies. This will be a more positivist data-driven approach to analysis. The causal analysis will focus more on searching for ‘nuggets of wisdom’ within the data [57]. Analysis will also draw on substantive formal theory to identify potential mechanisms and help make sense of recurring patterns in the data [49].

### Step 5. Further refine programme theories

Theory will be further refined in step 5 through realist theory testing qualitative interviews. The purpose of the theory testing interviews is to test, refine and refute the theories that have been developed through the realist review. The subject matter of the interview will be the programme theory, and the interviewees will help confirm, refute or refine the theory. This relationship between the interviewee and the interviewer is referred to as the teacher-learner-cycle [58]. The roles of teacher and learner are not static but fluid throughout the interview. Purposive sampling will be used to recruit 15 stakeholders involved in the funding and delivery of NBPs who have knowledge relevant to the programme theories. Interviewees from the initial theory gleaning interviews may be invited back to the theory testing interviews if they have knowledge relevant to the programme theory that is being tested. The interviews will take place online using Microsoft Teams and will last between 30 and 60 min. Informed consent will be sought prior to the start of the interview and all data will be anonymised.

## Dissemination

Results from the research project will be submitted to a peer-reviewed journal. The research project will follow the RAMESES publication standards [47] and RAMESES quality standards for realist reviews [59]. The refined programme theory that will emerge through the research process will be used to develop funding, targeting and recruitment guidelines for NBPs so that NBPs can be universally accessible. These guidelines will describe the relationship between funding of NBPs and targeting and recruitment strategies and the contexts within which they do/do not work. This will be of benefit to the organisations involved in the delivery of NBPs, funders of NBPs and the communities who access NBPs.

## Data Availability

Data will not be shared due to the risk of de-anonymisation.

## Appendix

**Table:** Initial programme theories to be tested

Level	Situation	If	Then	Because	Secondary outcomes	Necessary conditions
**Infrastructural**	Some people who would benefit from NBPs are excluded from attending the programme due to geographical variation in funding	If there is more funding for NBPs that are being delivered with areas experiencing greater structural disadvantage	Then people from disadvantaged areas are more likely to be able to access the NBP	Because the time and travel costs associated with attending a NBP will be lower The funding will enable providers to address barriers to implementation People recognise the NBP is for people like them	Impact on local health inequalities though the provision of an equigenic resource	For this to happen, there needs to be: Availability of accessible green and blue spaces Programme providers and facilitators able to deliver in disadvantaged areas
	Funders are concerned with ‘value for money’/numbers of people attending	If funders commit to funding NBPs that have lower levels of population impact but greater levels of individual impact	Then stakeholders involved in the design and delivery of NBPs can design NBPs that are targeted at specific communities	Because programme providers are likely to respond to the preferences of funders and specifically targeted programmes are likely to have greater levels of individual impact Monitoring and evaluation metrics will reflect the allowance for specificity and targeting. This will reassure providers that they can report only reaching a small number of people Providers will improve practice to seek the competitive funding	Programme staff have less pressure to get large numbers of recipients to the NBP so they can spend time deciding whether the NBP is appropriate for the recipient and gatekeep if necessary	Funding governing bodies need to be convinced that targeted work is important and that substantial work with smaller numbers of people can have a big impact
	For NBPs to be appropriate for the communities they are delivered in they must have community buy in	If local grassroots organisations are funded to deliver NBPs	The NBP will be more accessible to local communities	Local grassroots organisations are better able to tailor the programme to community needs and demographics Embedded in the community so have good links with community members Setting is familiar	Supply will meet demand for the programme as the local grassroots organisations are aware of the gaps in provision	Grassroots organisations need to be able to compete more fairly with larger organisations for funding and commissioning Grassroots organisations can play a key role in designing and targeting the NBP Sustainable funding models for small-scale grassroots organisations
**Institutional**	There is a lack of consensus around how community-based interventions such as NBPs can address health inequalities	If funders and commissioners of NBPs value/prioritise the use of prospective health/equity assessment tools to design and commission NBPs	Then funding and programmes will have evidence and justification to target communities/areas with greater need	Nature-based organisations involved in providing NBPs will respond to the preferences of funders to secure funding for the programme	Health equity tools will allow stakeholders to understand potential gaps in provision/areas where health inequalities may arise. Risks associated with health inequalities and NBPs will be accounted for in the design and delivery of the NBP. Therefore, local health inequalities are not made worse through the provision of individualised interventions	Stakeholders involved in the design, delivery and funding of NBPs need to be aware that there are equity assessment tools available to use and how to use them
	Funding is short term and prioritises novel interventions Relationships take time to build Understanding communities needs takes time	If funders are committed to funding projects that are longer term (e.g. 2 years plus)	Then stakeholders developing/delivering the programme have resources and time to build relationships, awareness and trust with local community groups	Because it can take time to understand gaps in local service provision and to find and engage with key members of local communities It can take time to tailor a programme to the needs of the community Short-term funding cycles mean that programme staff need to spend time frequently applying for funding rather than focus on the programme	Communities who typically are less likely to engage with individualised interventions may be more likely to engage with the NBP	For this to happen, funding needs to be sustainable for a long period of time. This can happen through larger providers creating sustainable funding models, or funders be willing to invest long term in small providers, and fund development phases and core capacity of organisations
**Individual**	NBPs vary in the level of engagement required	If the NBP is a rolling drop-in session	Then recipients will be more likely to attend	Because the programme requires less of a commitment/the programme is less daunting	There will be greater uptake of the NBP	For this to happen, providers need to have sufficient resources to provide regular drop-in sessions Good links with local community groups need to be established to ensure people are aware of the sessions
	Some people are anxious about attending NBPs/face social and cultural barriers	If the NBP is targeted at a specific community	People from the targeted community will be more likely to attend	They see that the programme is for people like them Less perceived risk or actual risk of feeling stigmatised The programme can be tailored to meet the specific needs of that community	If targeted at a community who are more likely to experience health inequalities, then there could be an impact on local health inequalities Increased perceived access for specific group of people	Providers and funders need to prioritise targeting the NBP
